# Cost-Effectiveness Analysis of Follow-Up Schedule for Hepatocellular Carcinoma after Radiofrequency Ablation

**DOI:** 10.1155/2022/3569644

**Published:** 2022-03-19

**Authors:** Shuifang Hu, Xiaoxue Wu, Mengchao Wei, Yunyan Ling, Meiyan Zhu, Yan Wang, Yong Chen, Meng Jin, Zhenwei Peng

**Affiliations:** ^1^Department of Radiation Oncology, The First Affiliated Hospital of Sun Yat-sen University, Guangzhou 510080, China; ^2^Department of Liver Surgery, The First Affiliated Hospital of Sun Yat-sen University, Guangzhou 510060, China; ^3^Clinical Trials Unit, The First Affiliated Hospital of Sun Yat-sen University, Guangzhou 510080, China; ^4^Institute of Precision Medicine, The First Affiliated Hospital of Sun Yat-sen University, Guangzhou 510080, China; ^5^Cancer Center, The First Affiliated Hospital of Sun Yat-sen University, Guangzhou 510080, China

## Abstract

**Methods:**

A Markov model was established to evaluate the cost-effectiveness of every 2 months or 2-3 months (2- to 3-month group) versus every 3 months or 3-4 months (3- to 4-month group) posttreatment surveillance in the first two years for HCC after RFA. Transition probabilities and utility values were derived from the literature review. Costs of follow-up were estimated from our institution. The incremental cost-effectiveness ratio (ICER), which was less than $10888 per quality-adjusted life-year (QALY), was considered cost-effective. Sensitivity analyses were performed to determine the uncertainty of the model.

**Results:**

The 2- to 3-month group gained 1.196 QALYs at a cost of $2212.66, while the effectiveness and cost of the 3- to 4-month group were 1.029 QALYs and $1268.92, respectively. The ICER of the 2- to 3-month group versus the 3- to 4-month group was $5651.14 per QALY gained, which was less than the willingness-to-pay threshold of 1-time gross domestic product per capita of China ($10888/QALY). One-way sensitivity analysis showed that the model was most sensitive to the utility of progression-free survival. The probabilistic sensitivity analysis demonstrated that the 2- to 3-month group had a higher probability of being more cost-effective than the 3- to 4-month group when willingness to pay was over $1088.8.

**Conclusions:**

Every 2 months or 2-3 months of follow-up intervals were more cost-effective than 3 months or 3-4 months of follow-up intervals. Thus, the intensive follow-up interval in the first two years was recommended for Child-Pugh class A or B HCC patients within the Milan criteria following RFA.

## 1. Introduction

Liver cancer is the sixth most common cancer and the third leading cause of cancer death worldwide in 2020, with approximately 906,000 new cases and 830,000 deaths [1]. Hepatocellular carcinoma (HCC) is the most common type of primary liver cancer, accounting for 75%–85% of total cases. With changes in aetiological and clinical features of HCC patients and wider use of surveillance during the last two decades, an increasing proportion of HCC patients diagnosed at an early stage are qualified for curative treatments such as radiofrequency ablation (RFA), contributing to the improved survival outcomes [2, 3] RFA, a widely used minimally invasive local therapy, has been shown to be an effective and safe strategy for treating early-stage HCC recommended by current guidelines [4–6]. Furthermore, RFA potentially provides similar therapeutic effects to liver resection for tumors between 3 and 5 cm in randomized controlled trials [7, 8]. However, up to 30% of patients develop tumor recurrence within two years following RFA with complete remission [9, 10]. Therefore, surveillance for early detection of recurrence after RFA therapy is a significant approach for long-term survival and better quality of life [9].

Laboratory tests such as serum alpha-fetoprotein and liver function, as well as imaging examinations including contrast-enhanced computed tomography (CT), magnetic resonance imaging (MRI), and contrast-enhanced ultrasound (CEUS), are recommended to monitor recurrence for HCC patients after radical therapy in current guidelines. Nevertheless, there remains no definite consensus on the follow-up schedule after RFA. For instance, the European Society for Medical Oncology Clinical Practice Guidelines report that HCC patients undergoing radical treatment should undertake dynamic CT or MRI every three months during the first year and every sixth months thereafter for early detection of recurrence [6], while in recent National Comprehensive Cancer Network Guidelines, multi-phase abdominal MRI or multi-phase CT scans were suggested to be performed at 3- to 6-month intervals within the first two years and then at 6∼12 months for surveillance following radical therapy [11]. According to the Asia-Pacific Clinical Practice Guidelines on the management of HCC, dynamic CT or MRI was recommended to be performed at 3-month intervals for at least 1 year and at 4- to 6-month intervals thereafter if there has been no relapse of tumor after one year [5]. Additionally, some previous studies reported a two-month follow-up interval for patients with HCC in the first two years following RFA treatment [7, 12, 13].

A previous study demonstrated that 3-monthly intervals can prolong survival when compared to 6-monthly intervals during the first year for HCC patients after curative treatment [14]. Excessively intensive follow-up may provide opportunities for early identification of recurrence and timely treatment but also lead to increasing consumption of medical costs. Therefore, we aimed to compare the cost-effectiveness of different follow-up modalities for early-stage HCC patients treated with RFA and achieve complete tumor response by developing a Markov analytic model.

## 2. Materials and Methods

### 2.1. Search Strategy

A systematic retrieval of PubMed, EMBASE, Web of Science, and the Cochrane Library was conducted from January 1, 2000, to December 31, 2020. The following terms were used in search: hepatocellular carcinoma, HCC, hepatic neoplasms, hepatic tumor, hepatic malignancy, liver neoplasms, liver cell carcinoma, liver cancer, radiofrequency ablation, RFA, ablation, and thermal ablation. The references of the included studies were also screened to identify additional studies. The included literature was divided into two groups according to the regular follow-up interval. Every 2 months or 2-3 months for a follow-up served as the 2- to 3-month group, and every 3 months or 3-4 months were regarded as the 3- to 4-month group. Because the data used in the decision analytic Markov model were derived from literature searches that did not involve identifiable patient information, the institutional review board approval was not necessary.

### 2.2. Eligibility Criteria

The inclusion criteria were as follows: (i) study object: patients diagnosed as HCC within the Milan criteria (a solitary tumor of ≤5 cm in diameter and up to 3 nodules of ≤3 cm in diameter) and treated with RFA; (ii) original research category: published retrospective study or randomized controlled trial; (iii) intervention measures: recurrence diagnosed during a regular surveillance visit based on imaging modalities such as CT, MRI, or contrast-enhanced ultrasound, and laboratory test included alpha-fetoprotein, liver function, and routine blood in the first two years; and (iv) outcome indicators: information on long-term survival. Exclusion criteria were as follows: (i) patients treated by RFA combined with other therapies; (ii) replicated data; (iii) incomplete outcome indicators; and (iv) case reports, abstracts, journal reviews, letters to the editor, and manuscript.

### 2.3. Data Extraction

Data extraction and analysis of all included studies were independently conducted by two researchers, and disagreements were resolved by discussion or referring to an experienced reviewer. Data not directly provided in the article were extracted from survival curves using Engauge Digitizer software v11.1. The following data were derived from the included studies: study design, number of patients, tumor size and number, Child-Pugh class, and median age.

The two-year recurrence-free survival (2-y RFS) or two-year disease-free survival obtained from the included studies for the 2- to 3-month group and the 3- to 4-month group was combined using the STATA version 16.0 and redefined as the 2-y RFS. Overall estimates of the 2-y RFS and the 95% confidence interval (CI) were calculated. Heterogeneity between different groups was measured through the *I*^2^ test, and a random-effects model was chosen for heterogeneous data.

### 2.4. Model Structure

**Figure 1:**
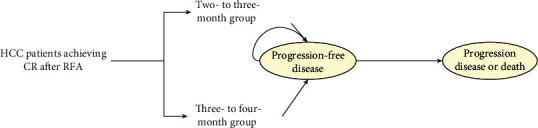
Abbreviated decision tree and Markov model. CR, complete remission; HCC, hepatocellular carcinoma; RFA, radiofrequency ablation.

A decision analytic Markov model using TreeAge Pro 2011 (TreeAge Software, Williams Town, MA) was conducted to evaluate the cost-effectiveness of follow-up strategy for HCC patients treated with RFA. Different follow-up strategies were compared in this model: every 2 months or 2-3 months (the 2- to 3-month group) or every 3 months or 3-4 months (the 3- to 4-month group) for a follow-up visit after RFA in the first two years. The Markov model included two health statuses: progression-free survival (PFS), and progression disease (PD) or death. All patients received regular posttreatment surveillance from progression-free survival to progression disease or death (Figure 1). PD or death was considered as the absorbing status. The Markov cycle length was one month, and the time horizon was 2 years. Constant monthly transition probabilities for each group were derived from the overall estimates of the 2-year RFS using the declining exponential approximation of life expectancy method [15].

### 2.5. Cost and Utility Estimate

Only direct medical costs associated with follow-up assessment were considered in this model, including examination costs for CT, alpha-fetoprotein, liver function, and routine blood. Cost estimates for each group were derived from our institution. Monthly costs for follow-up were $117.16 and $78.10 in the 2- to 3-month group and 3- to 4-month group, respectively. All costs were converted to US dollars according to $1 = RMB 6.56 in 2021. HCC utility value in the status of PFS (0.76) was extracted from the published literature [16].

### 2.6. Cost-Effectiveness Analysis

Costs and utilities were discounted at an annual rate of 5% [17]. The main output outcomes including expected total costs, quality-adjusted life-year (QALY), and incremental cost-effectiveness ratio (ICER) were obtained via the Markov model. The cost-effectiveness estimate of the two follow-up strategies was measured by ICER, representing the incremental cost per QALY gained. According to the World Health Organization evaluation criteria, a particular strategy was deemed cost-effective when the ICER was below the willingness-to-pay (WTP) threshold [18]. In this analysis, 1-time gross domestic product (GDP) per capita of China in 2020 was applied as the WTP threshold.

### 2.7. Sensitivity Analyses

Sensitivity analyses were used to explore the uncertainty of parameters. One-way and two-way sensitivity analyses were performed for all the key variables (Table 1) to show how each variable impacted the outcomes. New evaluation criteria and net monetary benefits (NMB) combining costs, effectiveness, and WTP were applied to reduce the mathematical uncertainty of ICER. The dominant strategy was chosen by higher NMB under the given WTP value. For health utilities, ranges of these parameters were based on the data obtained from the literature. For the transition probabilities and costs, by considering the lack of reported range data, a wider range of 50%–200% of the benchmark value was applied as described by Lim and colleagues [19]. Probabilistic sensitivity analysis using the Monte Carlo simulation of 10,000 repetitions was also performed, simultaneously varying all parameter values and their ranges. Costs were assigned using a gamma distribution, while utility values and probabilities were modeled using beta distributions.

## 3. Results

**Figure 2:**
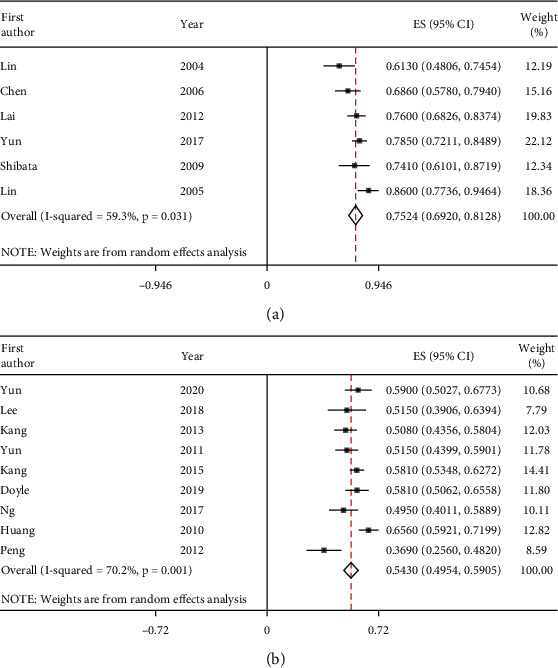
Forest plots of the study for each group. (a) The overall estimates of the 2-year recurrence-free survival (RFS) and the 95% confidence interval (CI) for HCC patients following radical radiofrequency ablation (RFA) in the 2- to 3-month group. (b) The overall estimates of the 2-y RFS and the 95% CI for HCC patients following radical RFA in the 3- to 4-month group.

A total of 15 references were included in the analysis, including four randomized controlled trials [7, 20–22] and two retrospective studies [12, 23] in the 2- to 3-month group and three randomized controlled trials [24–26] and six retrospective studies [10, 27–31] in the 3- to 4-month group. Of 1940 patients from the 15 included studies, 504 patients were classified into the 2- to 3-month group, and the rest of the 1436 patients were classified into the 3- to 4-month group. All the included patients had a liver function of Child-Pugh class A or B. The baseline characteristics of the eligible studies are summarized in Table 2. As shown in Figure 2, the pooled estimates of the 2-year RFS and 95% CI in the 2- to 3-month group (0.752 (95% CI: 0.692–0.813); *I*^2^ = 59.3%; *P* = 0.031), and the 3- to 4-month group (0.543 (95% CI: 0.495–0.591); *I*^2^ = 70.2%; *P* = 0.001) were calculated separately (Figure 2). The monthly transition probability of the 2- to 3-month group was 1.18%, and the corresponding value of the 3- to 4-month group was 2.51%.

### 3.1. Base Case Analysis

The base results of the cost-effectiveness analyses are presented in Table 3. The 2- to 3-month group yielded an average of 1.196 QALYs (14.35 months) with an additional 0.167 QALYs (2 months), compared with that of the 3- to 4-month group, which generated 1.029 QALYs (12.35 months). The costs for the 2- to 3-month group were $2212.66 and $1268.92 for the 3- to 4-month group. The ICER of the 2- to 3-month group versus the 3- to 4-month group was $5651.14 per QALY gained, which was much lower than the WTP threshold of one-time GDP per capita of China ($10888/QALY). This result indicated that every 2 months or 2-3 months for a posttreatment visit were more likely a cost-effective follow-up strategy for HCC patients after RFA.

### 3.2. One-Way Sensitivity Analysis

**Figure 3:**
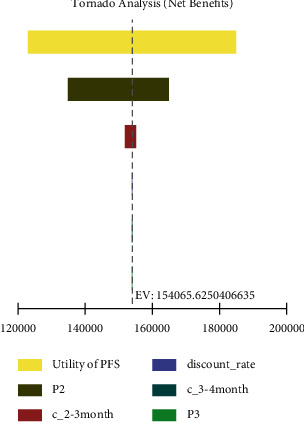
Tornado diagram for one-way sensitivity analyses. PFS, progression-free survival; PD, progression disease; P2, transition probability from PFS to PD in the 2- to 3-month group; c_2-3 months, costs of the 2- to 3-month group per circle; P3, transition probability from PFS to PD in the 3- to 4-month group; c_3-4 months, costs of the 3- to 4-month group per circle.

The tornado diagram of one-way sensitivity analyses is shown in Figure 3. The parameter with the most significant influence on the NMB was the utility of PFS. The NMB of the 2- to 3-month group was always larger than that of the 3- to 4-month group regardless of the utility of PFS (Supplementary Figure 1(a)), suggesting that the 2- to 3-month group was the dominant strategy over the range we tested. The results showed that NMB was also sensitive to variations of the transition probability from PFS to PD in the 2- to 3-month group and the costs of the 2- to 3-month group per cycle. When the transition probability from PFS to PD in the 2- to 3 -month group was less than 0.024 (Supplementary Figure 1(b)), the 2- to 3-month group remained more cost-effective than the 3- to 4-month group. Besides, despite the costs of the 2- to 3-month group per cycle changed within the range (Supplementary Figure 1(c)), the NMB of the 2- to 3-month group was always greater than that of the 3- to 4-month group, which indicated that the 2- to 3-month group was the dominant strategy. Other variables, such as discount rate, the costs of the 3- to 4-month group per cycle, and the transition probability from PFS to PD in the 3- to 4-month group, had only minor effects on the NMB.

### 3.3. Two-Way Sensitivity Analysis

**Figure 4:**
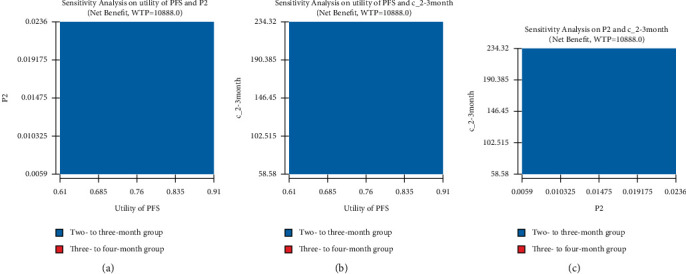
Two-way sensitivity analysis of the two- to three-month group and the three- to four-month group. (a) Two-way sensitivity analysis of P2 and utility of PFS for the NMB. (b) Two-way sensitivity analysis of c_2-3 months and the utility of PFS for the NMB. (c) Two-way sensitivity analysis of c_2-3 months and P2 for the NMB. PD, progression disease; P2, transition probability from PFS to PD in the 2- to 3-month group; PFS, progression-free survival; NMB, net monetary benefits; c_2-3 months, costs of the 2- to 3-month group per circle.

The top three sensitive variables were further analyzed in the two-way sensitivity analysis. The results demonstrated that every 2 months or 2-3 months for a follow-up visit were always the cost-effective surveillance strategy no matter how the utility of PFS, the transition probability from PFS to PD in the 2- to 3-month group, and the costs of the 2- to 3-month group per cycle varied within the set ranges of the parameters (Figure 4).

### 3.4. Probabilistic Sensitivity Analysis

**Figure 5:**
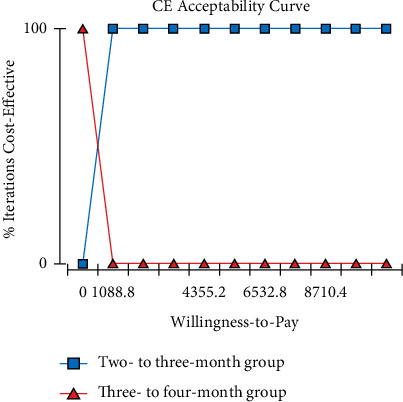
Cost-effectiveness acceptability curve for early-stage HCC patients following RFA. CE, cost-effectiveness; HCC, hepatocellular carcinoma.

The cost-effectiveness acceptability curves showed that the 2- to 3-month group had a higher probability of being more cost-effective than the 3- to 4-month group when WTP was over $1088.8 (Figure 5). Given the Chinese WTP threshold of $10888, the probability for the 2- to 3-month group to be cost-effective was 100%, and the corresponding value for the 3- to 4-month group was 0%.

## 4. Discussion

HCC patients undergoing RFA with complete remission were supposed to adopt regular follow-up for early detection of tumor recurrence and timely treatment. Clinical follow-up intervals ranged from two to six months in the first two years due to the absence of definite evidence regarding an optimal interval for posttreatment surveillance. An intensive follow-up strategy may benefit survival outcomes; however, medical costs increase simultaneously. Based on the results of base case and sensitivity analyses, our study demonstrated that every 2 months or 2-3 months for a follow-up visit were more cost-effective compared with 3 months or 3- to 4-month intervals for Child-Pugh class A or B HCC patients within the Milan criteria following RFA.

To date, several studies have explored the cost-effectiveness of follow-up modalities in different types of cancer including breast cancer, colorectal cancer, and lung cancer after radical therapy [32–36]. As shown in a previous study, increasing follow-up inspections including annual hepatic echography, chest X-ray, and bone scan did not enhance the detection rate of tumor relapses in patients with breast cancer after curative therapy [33]. Similarly, another study reported that shortening hospital follow-up time could remarkably reduce costs while did not affect the detection rate of tumors less than 2 cm after breast cancer treatment [32]. On the contrary, intensive follow-up schedules with more medical examinations and hospital visits had been economically justified for the increment of the detection of asymptomatic recurrences and improvement of all-cause mortality in colorectal cancer [34–36]. It can be seen that not all patients will benefit from intensive surveillance, and the appropriate strategies for posttreatment surveillance may vary in different malignancies. As for HCC treated with radical therapy, some studies have investigated the association between different follow-up strategies and survival outcomes; however, conclusions varied from studies to studies. Liu et al. [37] found that the overall survival of the short-interval group (<4 months) was better than that of the long-interval group (4–6 months) for HCC patients with multiple tumors less than 3 cm or a solitary tumor between 3 and 5 cm following curative thermal ablation. In contrast, the intensive interval for follow-up visits has not been shown to prolong overall survival as reported in two recent studies [38, 39]. However, costs of follow-up were not taken into account in these prior studies.

To the best of our knowledge, little was known on the cost-effectiveness analysis of follow-up intervals for HCC patients following curative treatment. Thus, the cost and effectiveness of follow-up for HCC patients after RFA were considered simultaneously in this study. Only direct medical costs for regular surveillance following RFA were considered in our model, including routine laboratory tests and imaging examinations such as dynamic CT, MRI, or CEUS. Liver biopsy was not included in the cost calculation for its invasiveness, although the risk of false-negative results based on noninvasive diagnosis such as CEUS may be high for different vascular patterns in small recurrent nodules (10-30 mm) after RFA, as previously reported [40]. Our findings demonstrated that the 2- to 3-month group produced 1.196 QALYs, whereas the 3- to 4-month group offered 1.029 QALYs. The effect of the 2- to 3-month group was better than that of the 3- to 4-month group, which was possibly due to intensive surveillance that can identify tumor relapse at an earlier time point. Hence, initial recurrent hepatocellular carcinoma patients can receive timely curative-intent retreatments, including repeat ablation, surgery, and liver transplantation. With respect to the CE analysis, we found that every 2 months or 2-3 months for a follow-up visit were more cost-effective compared with the 3-month or 3- to 4-month interval for HCC after RFA. The ICER of the 2- to 3-month group versus the 3- to 4-month group was $5651.14/QALY, which was significantly less than the WTP threshold of $10888/QALY, indicating that the 2- to 3-month group was more cost-effective. Therefore, intensive interval (2-month or 2- to 3-month interval) following RFA treatment was recommended for better CE. According to the tornado diagram, the utility of the PFS was the most sensitive parameter, which was reasonable for a higher utility value of PFS usually indicating a better survival outcome. Moreover, the value of the cross point of the CE acceptability curves was $1088.8, which was much lower than the given WTP. This finding suggested that the 2-month or 2- to 3-month follow-up strategies were the preferred choice for the majority of patients with affordable medical costs in most scenarios. The 3- to 4-month group may be accepted for the minority of subjects with rather poor economic conditions when the WTP was lower than $1088.8.

Our study had several limitations. First, the costs of follow-up estimated in our model were derived from our institution without considering the price differences in different regions. Thus, a wide range of costs (50%–200% of benchmark value) was used in the sensitivity analyses to reduce the uncertainty in the model. Moreover, the result of ICRE was compared with one-time GDP per capita of China as the WTP threshold instead of other countries to minimize the bias. Second, the Engauge Digitizer used to extract survival outcomes from the survival curves may produce unavoidable data deviation. Third, theoretically, HCC patients with risk factors for recurrence such as hepatitis virus infections might benefit more from intensive follow-up strategy after curative treatment. A meta-analysis reported that recurrence and survival probabilities are extremely variable in hepatitis C virus-related early HCC patients after curative ablation and follow-up duration are associated with higher recurrence [41]. However, our analysis was not stratified according to risk factors for recurrence due to insufficient corresponding reports, which is worthy of further investigation.

In conclusion, every 2 months or 2-3 months for a posttreatment visit were more cost-effective compared with every 3 months or 3-4 months for Child-Pugh class A or B HCC patients within the Milan criteria after RFA. However, further high-quality studies are warranted to validate our findings.

## Figures and Tables

**Table 1:** Base parameter input to model and ranges of sensitivity analysis.

Parameters	Baseline value	Range	Distribution	Source
*Transition probabilities (monthly)*				
PFS to PD in the 2- to 3-month group	1.18%	0.59–2.36%	Beta	
PFS to PD in the 3- to 4-month group	2.51%	1.26–5.02%	Beta	

*Costs per circle ($)*				
Two-month group monthly cost	117.16	58.58–234.32	Gamma	
Three-month group monthly cost	78.10	39.05–156.20	Gamma	

*Utility value*				
Utility PFS	0.76	0.61–0.91	Beta	[15]
Utility PD	0.68	0.54–0.82	Beta	[15]
*Discount rate*	5%	0%–5%	Fixed	[16]

PD, progressive disease; PFS, progression-free survival.

**Table 2:** Baseline characteristics of the included studies.

Published year	First author	Study design	No. of patients	Inclusion criteria in tumor size and tumor number	Child-Pugh class	Interval of follow-up	Age, years, median (range), or mean ± SD
2011	Yun	Retro	170	Single tumor ≤3 cm	Child A5	3 months	57.0 ± 9.9
2013	Kang	Retro	183	Single tumor ≤3 cm	Child A/B	3 months	57.2 ± 9.4
2015	Kang	Retro	438	Single tumor ≤3 cm	Child A/B	3 months	58 (30–80)
2018	Lee	Retro	62	Single tumor ≤3 cm	Child A/B	3 months	56.0 ± 9.7
2019	Doyle	Retro	167	Single tumor ≤2 cm	Child A/B	3 months	57.6 ± 6.0
2020	Yun	Retro	122	Milan criteria	Child A/B	3 months	69.1 ± 10.1
2010	Huang	RCT	115	Milan criteria	Child A/B	3 months	54.7 ± 12.2
2017	Ng	RCT	109	Milan criteria	Child A/B	3 months	57 (23–78)
2012	Peng	RCT	70	Milan criteria (recurrence)	Child A/B	3-4 months	55.1 ± 9.5
2012	Lai	Retro	117	Single tumor ≤3 cm	Child A/B	2 months	54.9 ± 11.3
2004	Lin	RCT	52	Size ≤4 cm and number ≤3	Child A/B	2 months	62.0 ± 11.0
2006	Chen	RCT	71	Single tumor ≤5 cm	Child A	2 months	51.9 ± 11.2
2005	Lin	RCT	62	Size ≤3 cm and number ≤3	Child A/B	2-3 months	61.0 ± 10.0
2009	Shibata	RCT	43	Size ≤3 cm and number ≤3	Child A/B	2-3 months	69.8 ± 8.0
2017	Yun	Retro	159	Single tumor ≤2 cm	Child A/B	2-3 months	54.0 ± 11.0

RCT, randomized controlled trial; Retro, retrospective study.

**Table 3:** Summary of cost and effectiveness results in scenario.

Strategy	Effect, QALY	Incremental effect	Cost, $	Incremental cost	ICER, $/QALY
Three- to four-month group	1.029	0	1268.92	0	0
Two- to three-month group	1.196	0.167	2212.66	943.74	5651.14

ICER, incremental cost-effectiveness ratio; QALY, quality-adjusted life-years.

## Data Availability

The data that support the findings of this study are openly available in previously published references.

## Abbreviations:

CI: Confidence interval
HCC: Hepatocellular carcinoma
ICER: Incremental cost-effectiveness ratio
NMB: Net monetary benefits
PD: Progression disease
PFS: Progression-free survival
QALY: Quality-adjusted life-year
RFA: Radiofrequency ablation
RFS: Recurrence-free survival
WTP: Willingness to pay.

## Supplementary Materials

Supplementary Materials Supplementary Figure 1: one-way sensitivity analysis of the “two- to three-month group and the three- to four-month group. (a) One-way sensitivity analysis of the utility of PFS for the NMB. (b) One-way sensitivity analysis of P2 for the NMB. (c) One-way sensitivity analysis of c_2-3 months for the NMB. PD, progression disease; P2, transition probability from PFS to PD in the 2- to 3-month group; PFS, progression-free survival; NMB, net monetary benefits; c_2-3 months, costs of the 2- to 3-month group per circle.
