# Supplementary figures and images for: Cost-Effectiveness Analysis of Follow-Up Schedule for Hepatocellular Carcinoma after Radiofrequency Ablation
Source: J Oncol. 2022 Mar 19;2022:3569644. doi: 10.1155/2022/3569644 (PMC8957434; doi:10.1155/2022/3569644)

A

## Sensitivity Analysis

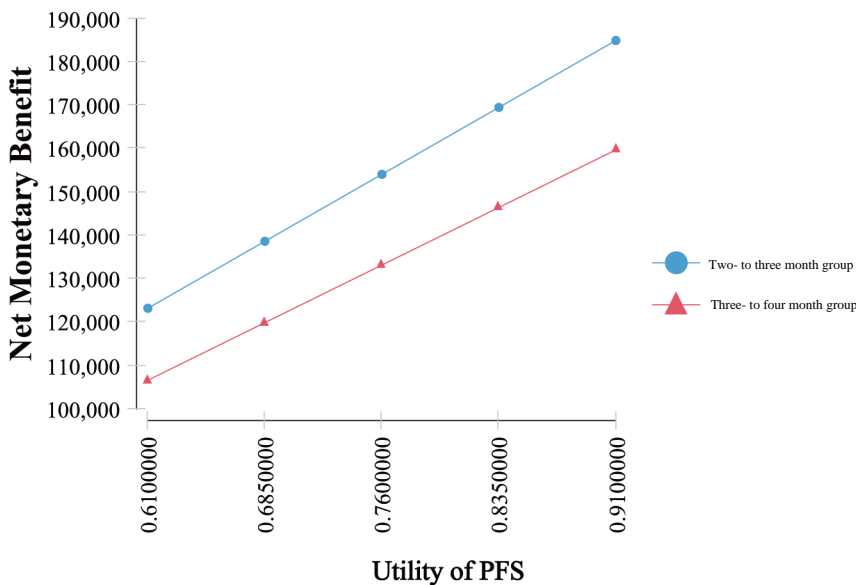

B

## Sensitivity Analysis

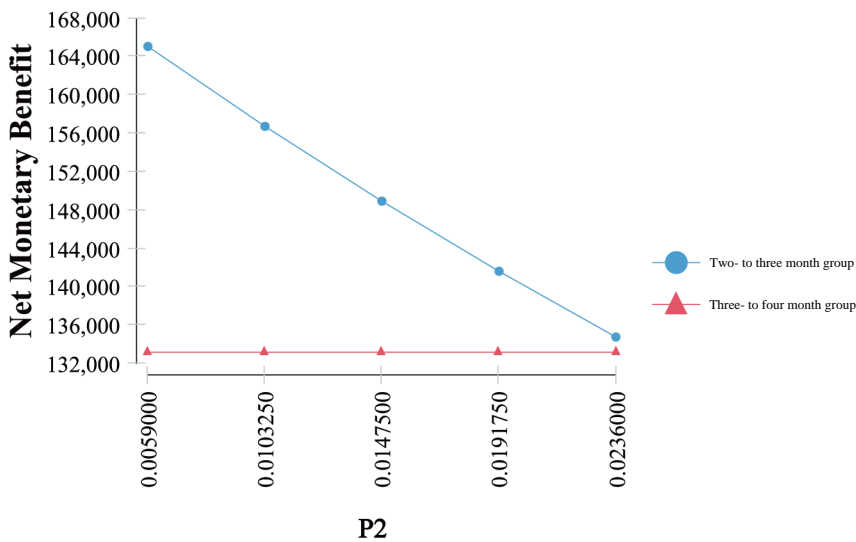

C

## Sensitivity Analysis

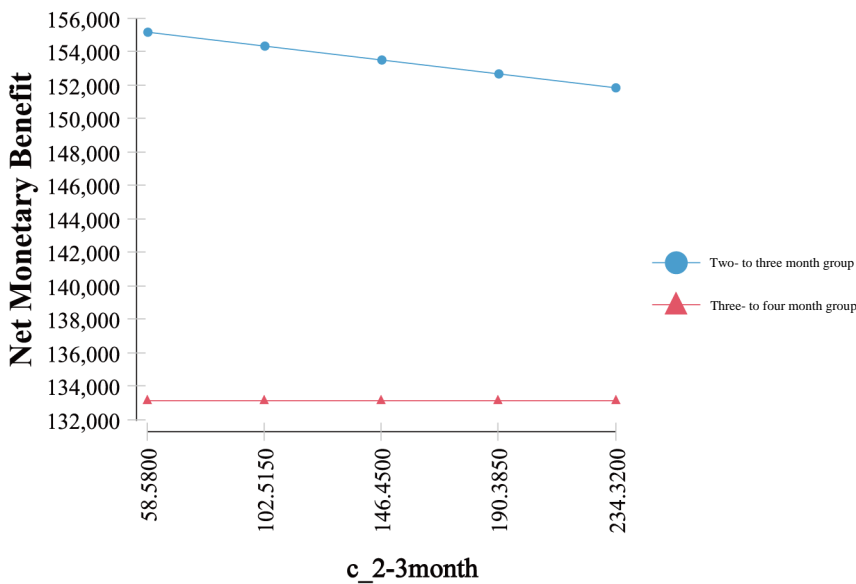

Supplement: Supplementary Materials — Supplementary Figure 1: one-way sensitivity analysis of the “two- to three-month group and the three- to four-month group. (a) One-way sensitivity analysis of the utility of PFS for the NMB. (b) One-way sensitivity analysis of P2 for the NMB. (c) One-way sensitivity analysis of c_2-3 months for the NMB. PD, progression disease; P2, transition probability from PFS to PD in the 2- to 3-month group; PFS, progression-free survival; NMB, net monetary benefits; c_2-3 months, costs of the 2- to 3-month group per circle. [file 3569644.f1.pdf]
